# A multimodal X-ray spectroscopy investigation of uranium speciation in ThTi_2_O_6_ compounds with the brannerite structure

**DOI:** 10.1038/s41598-023-38912-1

**Published:** 2023-08-07

**Authors:** Malin C. Dixon Wilkins, Luke T. Townsend, Martin C. Stennett, Kristina O. Kvashnina, Claire L. Corkhill, Neil C. Hyatt

**Affiliations:** 1 Department of Materials Science and Engineering, University of Sheffield, Sheffield, UK; 2 School of Mechanical and Materials Engineering, Washington State University, Pullman, WA 99164 USA; 3 The Rossendorf Beamline at ESRF, CS 40220, 38043 Grenoble Cedex 9, France; 4 Institute of Resource Ecology, Helmholtz-Zentrum Dresden-Rossendorf, Bautzner Landstrasse 400, 01328 Dresden, Germany; 5 School of Earth Sciences, The University of Bristol, Bristol, BS8 1RL UK

**Keywords:** Nuclear chemistry, Nuclear waste

## Abstract

ThTi_2_O_6_ derived compounds with the brannerite structure were designed, synthesised, and characterised with the aim of stabilising incorporation of U^5+^ or U^6+^, at dilute concentration. Appropriate charge compensation was targeted by co-substitution of Gd^3+^, Ca^2+^, Al^3+^, or Cr^3+^, on the Th or Ti site. U L_3_ edge X-ray Absorption Near Edge Spectroscopy (XANES) and High Energy Resolution Fluorescence Detected U M_4_ edge XANES evidenced U^5+^ as the major oxidation state in all compounds, with a minor fraction of U^6+^ (2–13%). The balance of X-ray and Raman spectroscopy data support uranate, rather than uranyl, as the dominant U^6+^ speciation in the reported brannerites. It is considered that the U^6+^ concentration was limited by unfavourable electrostatic repulsion arising from substitution in the octahedral Th or Ti sites, which share two or three edges, respectively, with neighbouring polyhedra in the brannerite structure.

## Introduction

The mineral brannerite, prototypically UTi_2_O_6_^1^, is of economic importance in production of uranium for the nuclear fuel cycle^2^. Although typically metamict due to self-radiation damage, natural brannerite specimens retain significant fractions of their actinide inventory under geochemical conditions over geological timescales (10^7^–10^8^ Ma)^3–6^. Indeed, brannerite is known to be amongst the most refractory of uranium minerals, highly resistant to leaching under acidic conditions, but potentially less resistant to dissolution under alkaline conditions^7,8^. Consequently, UTi_2_O_6_ is of interest as a host phase for the immobilisation of long lived actinides, such as plutonium, in tailored ceramic and glass–ceramic composite materials, as radioactive wasteforms^9–21^.

Taking the general formula AB_2_O_6_, the monoclinic brannerite structure, characteristic of UTi_2_O_6_, comprises corrugated sheets of edge sharing BO_6_ octahedra connected by chains of edge sharing AO_6_ octahedra, in space group *C2/m*^2^. This structure is also adopted by the high temperature polymorph of ThTi_2_O_6_, synonymous with the mineral species thorutite^22^. Several U^5+^ dominant brannerites have been previously reported, typically stabilised by substitution of trivalent lanthanides on the U site (*i.e.* (U^5+^_0.5_Ln^3+^_0.5_)Ti_2_O_6_, Ln^3+^ including Gd^3+^, Ce^3+^, Dy^3+^, Tb^3+^, etc*.*)^9–14^. Recently, we reported a novel U^5+^ dominant brannerite, U_1.09_(Ti_1.29_Al_0.71_)O_6_, stabilised by an alternative crystal chemical strategy, involving Al^3+^ substitution on the Ti site^17^. Whilst the stability of U^4+^ and U^5+^ species within the brannerite structure is well-established, the stability of U^6+^ species is less clear, but evidently important to fully understand the geochemical stability of brannerite minerals, and the design and performance of their synthetic wasteform counterparts.

Previous investigation by Zhang et al*.* examined the compounds (Th_0.85_U_0.10_Ca_0.05_)Ti_2_O_6_ (targeting U^5+^ only) and (Th_0.90_U_0.05_Ca_0.05_)Ti_2_O_6_ (targeting U^6+^ only), based on ThTi_2_O_6_, with the brannerite structure^15,16^. The incorporation of uranyl species, (UO_2_)^2+^, was inferred based on the observation of weak bands at 780.9 and 807.8 cm^−1^, in the respective Raman spectra (attributed as the ν_*1*_ symmetric stretch). However, no *direct determination* of uranium oxidation state, for example by X-ray absorption or photoelectron spectroscopy, was reported. Finnie et al*.* investigated (Th_0.55_U_0.30_Ca_0.15_)Ti_2_O_6_, and established U^5+^ as the dominant oxidation state (75%), with minor U^4+^ (15%) and U^6+^ (10%), using X-ray photoelectron spectroscopy (XPS)^18^. The U^6+^ contribution was considered to be a consequence of surface oxidation of the brannerite material, combined with the surface sensitivity of XPS. Interestingly, a higher fraction of U^4+^ was determined from XPS data acquired from a polished surface of (Th_0.55_U_0.30_Ca_0.15_)Ti_2_O_6_, compared to a fracture surface, which was attributed to enrichment of Ca^2+^ and U^5+^ and/or U^6+^ at grain boundaries.

To further elucidate the incorporation and stability of U^6+^ in the brannerite structure, we designed three solid solutions based on ThTi_2_O_6_, targeting U^5+^ or U^6+^ speciation, with charge compensation by co-substitution on the Th or Ti site:

- (Th_0.85_U^5+^_0.10_Ca_0.05_)Ti_2_O_6_ and (Th_0.90_U^6+^_0.05_Ca_0.05_)Ti_2_O_6_
- (Th_0.80_U^5+^_0.10_Gd_0.10_)Ti_2_O_6_ and (Th_0.85_U^6+^_0.05_Gd_0.10_)Ti_2_O_6_
- (Th_0.95_U^5+^_0.05_)(Ti_1.95_M_0.05_)O_6_ and (Th_0.95_U^6+^_0.05_)(Ti_1.90_M_0.10_)O_6_; with M^3+^  = Al^3+^, Cr^3+^

Two additional compositions were also examined, reflecting the substitution of U for Th in the same proportions as the above compositions, without charge compensating species:

- (Th_0.95_U_0.05_)Ti_2_O_6_ and (Th_0.90_U_0.10_)Ti_2_O_6_

Recognising that ThTi_2_O_6_ does not present redox flexibility when prepared under oxidising conditions, our intent was to control uranium speciation as the most significant variable in each solid solution, by judicious co-substitution of appropriate charge compensating species with known oxidation state. Charge compensating species were chosen based on their previously reported solid solubilities in the brannerite structure and/or similar titanate structures: Ca^2+^ and Gd^3+^ on the Th^4+^ site^9–11^; Al^3+^ and Cr^3+^ on the Ti^4+^ site^19,23,24^. Note that compositions (Th_0.85_U^5+^_0.10_Ca_0.05_)Ti_2_O_6_ and (Th_0.90_U^6+^_0.05_Ca_0.05_)Ti_2_O_6_ are nominally identical to those previously investigated by Zhang et al*.*, enabling a direct comparison, in principal, with this earlier study^16^.

Herein, we show that a minor fraction of U^6+^ (2–13%) was indeed stabilised within the synthesised brannerite compositions, by High Energy Resolution Fluorescence Detected (HERFD) X-ray Absorption Near Edge Spectroscopy (XANES) at the U M_4_ edge. However, both U L_3_ edge XANES and HERFD U M_4_ edge XANES evidenced U^5+^ as the major oxidation state in all compounds. The evidence in support of uranate and uranyl speciation, and the factors limiting the concentration of U^6+^, are discussed.

## Results

### X-ray diffraction

**Figure 1:**
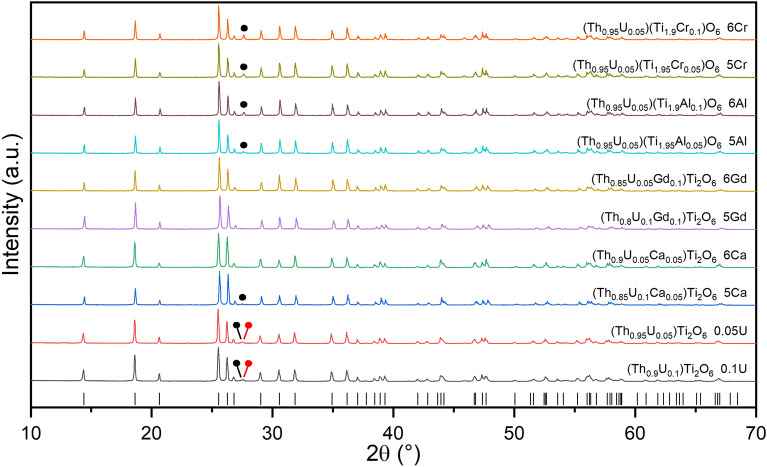
X-ray diffraction patterns of ThTi_2_O_6_ compositions adopting the brannerite structure, targeting U^5+^ and U^6+^ incorporation with appropriate charge compensation; nominal compositions are summarized in Table 1 and Table 2. Tick marks below show the reflection positions of ThTi_2_O_6_ (PDF #04-007-2825). Diagnostic reflections of ThO_2_ are marked with black circles; TiO_2_ (rutile) by red circles. Composition identifiers generally use the nomenclature *nEl*, where *n* is the target U oxidation state and *El* is the charge compensating element.

X-ray diffraction was used to characterise the phases present in each product, as shown in Fig. 1, and summarised in Table 1 and Table 2. A compound with the brannerite structure (ThTi_2_O_6_; PDF #04-007-2825) was the major phase formed in all compositions. Trace quantities of TiO_2_ (rutile) and ThO_2_ were also present in many compositions; trace ThO_2_ was differentiated from UO_2_ by electron microscopy observation of the microstructure and EDX analysis. No reflections characteristic of UO_2_, U_3_O_8_ or UO_3_ were observed; the estimated limit of detection of these oxides is around 0.5 wt% as determined by simulation of X-ray diffraction patterns. The diffraction patterns of compositions targeting Al^3+^ or Cr^3+^ substitution on the Ti^4+^site, exhibited reflections characteristic of trace ThO_2_. In contrast, the diffraction patterns of compositions targeting Ca^2+^ or Gd^3+^ substitution on the Th^4+^site did not generally exhibit reflections characteristic of trace ThO_2_.

**Table 1:** Unit cell parameters determined from LeBail analysis of X-ray diffraction patterns for ThTi_2_O_6_ compositions, adopting the brannerite structure, targeting U^5+^ and U^6+^ incorporation with appropriate charge compensation, together with selected literature data. A weighted average cation radius (*r*_*w*_) was calculated assuming nominal or reported compositions as appropriate, assuming U^5+^. R_wp_ and χ^2^ goodness-of-fit metrics from the Le Bail analysis are also included. Sample identification reference (ID) refers to Fig. 1 and generally uses the nomenclature *nEl*, where *n* is the target U oxidation state and *El* is the charge compensating element (or otherwise refers to a literature reference).

Nominal Composition	ID	a (Å)	b (Å)	c (Å)	β (°)	Volume (Å^3^)	R_wp_	GOF	*r*_*w*_ (Å)
(Th_0.90_U_0.10_)Ti_2_O_6_	0.1U	9.8133(3)	3.81475(8)	7.0226(2)	118.780(1)	230.420(10)	9.39	1.51	0.8506
(Th_0.95_U_0.05_)Ti_2_O_6_	0.05U	9.8092(2)	3.81617(6)	7.0232(1)	118.786(1)	230.416(8)	9.77	1.57	0.8536
(Th_0.85_U_0.10_Ca_0.05_)Ti_2_O_6_	5Ca	9.8174(2)	3.80461(7)	7.0087(1)	118.775(1)	229.456(9)	12.88	1.20	0.8516
(Th_0.90_U_0.05_Ca_0.05_)Ti_2_O_6_	6Ca	9.8187(2)	3.81439(7)	7.0220(1)	118.801(1)	230.458(9)	13.60	1.20	0.8546
(Th_0.80_U_0.10_Gd_0.10_)Ti_2_O_6_	5Gd	9.8164(2)	3.79973(6)	7.0024(1)	118.765(1)	228.956(8)	9.66	1.07	0.8506
(Th_0.85_U_0.05_Gd_0.10_)Ti_2_O_6_	6Gd	9.8221(2)	3.80485(8)	7.0102(2)	118.789(1)	229.600(9)	11.32	1.08	0.8536
(Th_0.95_U_0.05_)(Ti_1.95_Al_0.05_)O_6_	5Al	9.8114(2)	3.81346(6)	7.0170(1)	118.835(1)	229.988(7)	10.93	1.07	0.8525
(Th_0.95_U_0.05_)(Ti_1.90_Al_0.10_)O_6_	6Al	9.8107(2)	3.81329(6)	7.0163(1)	118.834(1)	229.944(7)	10.65	1.07	0.8513
(Th_0.95_U_0.05_)(Ti_1.95_Cr_0.05_)O_6_	5Cr	9.8163(2)	3.81668(7)	7.0220(1)	118.824(1)	230.487(8)	11.03	1.05	0.8538
(Th_0.95_U_0.05_)(Ti_1.9_Cr_0.10_)O_6_	6Cr	9.8170(2)	3.81633(5)	7.0216(1)	118.837(1)	230.441(6)	10.40	1.07	0.8540
ThTi_2_O_6_	^26^	9.8140(2)	3.8228(1)	7.0313(2)	118.82(1)	231.12*	–	–	0.8567
UTi_2_O_6_	^8^	9.8123(15)	3.7697(6)	6.9253(9)	118.957(6)	224.14*	–	–	0.8400
(U_0.54_Y_0.46_)Ti_2_O_6_	^27^	9.8039(2)	3.7188(1)	6.8403(2)	118.52(1)	219.12(1)	–	–	0.8241
(U_0.74_Ca_0.26_)Ti_2_O_6_	^11^	9.8008(2)	3.7276(1)	6.8745(1)	118.38(1)	220.97(1)	–	–	0.8175
(U_0.50_Tb_0.50_)Ti_2_O_6_	^12^	9.808(2)	3.725(4)	6.871(9)	118.51*	220.6(1)	–	–	0.8238
(U_0.50_Dy_0.50_)Ti_2_O_6_	^12^	9.810(2)	3.723(4)	6.857(8)	118.49*	220.1(1)	–	–	0.8220
(U_0.49_Y_0.51_)Ti_2_O_6_	^13^	9.8086(6)	3.7179(3)	6.8418(5)	118.52(6)	219.2(1)	–	–	0.8205

*Calculated from literature reports of the unit cell parameters.

**Table 2:** Tabulated information from characterisation of ThTi_2_O_6_ compositions, adopting the brannerite structure, targeting U^5+^ and U^6+^ incorporation with appropriate charge compensation (see Table 1). The secondary phases identified in the X-ray diffraction patterns and U oxidation states, as-determined by linear regression (LR) of the U L_3_ edge position, and linear combination fitting (LCF) and iterative target transformation factor analysis (ITFA) of HERFD U M_4_ edge XANES spectra, are also included.

Target brannerite composition	ID	Target U ox. State	Secondary phases	U ox. state L_3_ edge LR	U ox. state M_4_ edge LCF	U ox. state M_4_ edge ITFA
(Th_0.90_U_0.10_)Ti_2_O_6_	0.1U	–	TiO_2_, ThO_2_	5.0(2)+	4.93(12)+	4.91(6)+
(Th_0.95_U_0.05_)Ti_2_O_6_	0.05U	–	TiO_2_, ThO_2_	5.0(2)+	4.91(12)+	4.89(6)+
(Th_0.85_U_0.10_Ca_0.05_)Ti_2_O_6_	5Ca	5+	ThO_2_	5.0(2)+	4.98(9)+	4.96(6)+
(Th_0.90_U_0.05_Ca_0.05_)Ti_2_O_6_	6Ca	6+	–	5.0(2)+	5.06(9)+	5.04(6)+
(Th_0.80_U_0.10_Gd_0.10_)Ti_2_O_6_	5Gd	5+	Trace TiO_2_/ThO_2_	4.9(2)+	5.06(7)+	5.04(6)+
(Th_0.85_U_0.05_Gd_0.10_)Ti_2_O_6_	6Gd	6+	Trace TiO_2_	5.1(2)+	5.12(11)+	5.11(6)+
(Th_0.95_U_0.05_)(Ti_1.95_Al_0.05_)O_6_	5Al	5 +	ThO_2_	4.8(2)+	5.04(9)+	5.02(6)+
(Th_0.95_U_0.05_)(Ti_1.90_Al_0.10_)O_6_	6Al	6+	ThO_2_	4.8(2)+	5.10(13)+	5.09(6)+
(Th_0.95_U_0.05_)(Ti_1.95_Cr_0.05_)O_6_	5Cr	5 +	ThO_2_	4.9(2)+	5.00(11)+	4.98(6)+
(Th_0.95_U_0.05_)(Ti_1.90_Cr_0.10_)O_6_	6Cr	6+	ThO_2_	5.1(2)+	5.04(10)+	5.02(6)+

### Unit cell parameters

**Figure 2:**
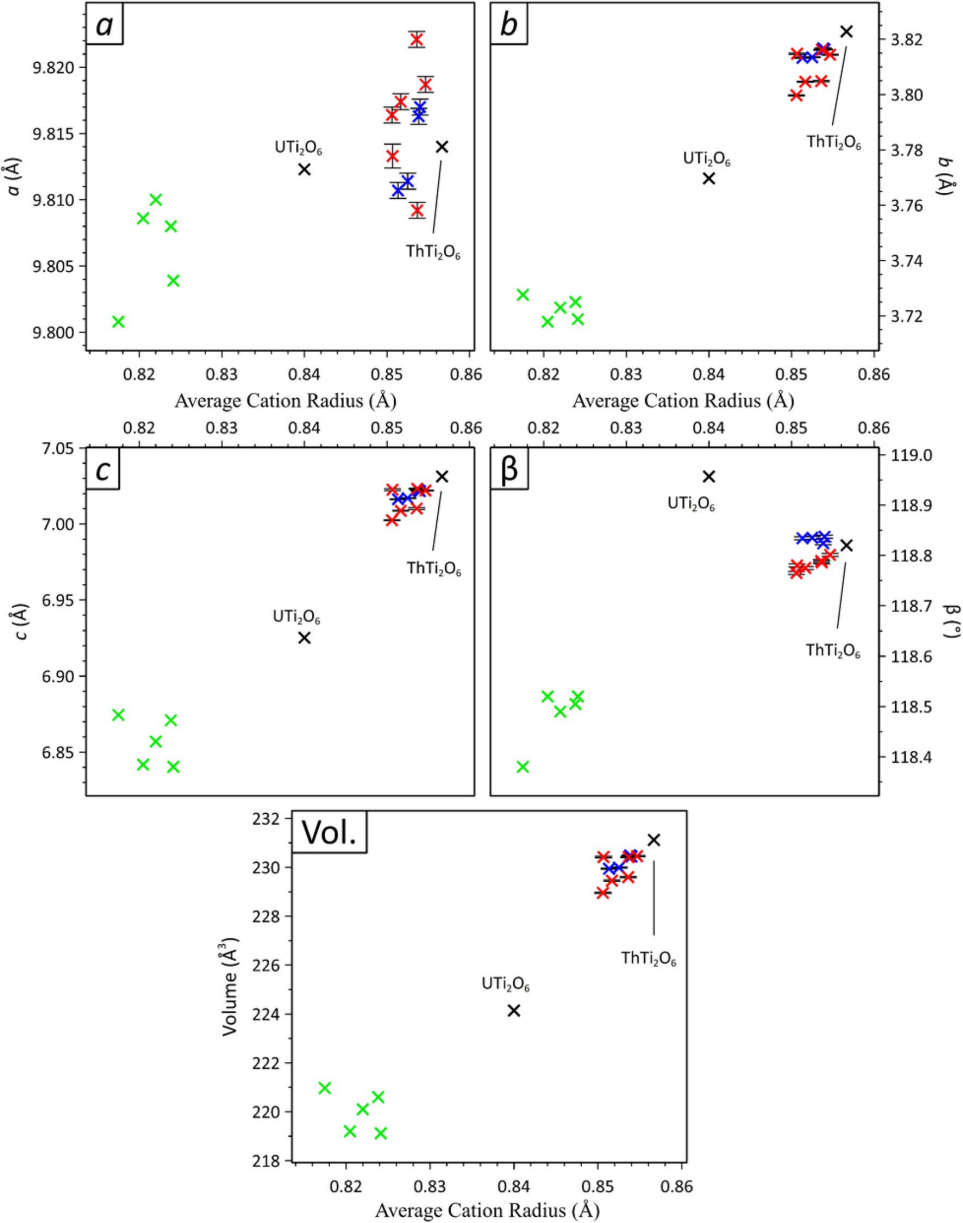
Plots of unit cell parameters, angle β, and volume, as a function of weighted average cation radius of compounds with the brannerite structure; data for ThTi_2_O_6_ and UTi_2_O_6_ are shown as black crosses; data from this study for ThTi_2_O_6_ compositions targeting U^5+^ or U^6+^ incorporation, with appropriate charge compensation, are shown as red (Th site) and blue (Ti site) symbols; data for U^5+^ brannerites (derived from UTi_2_O_6_) are shown as green symbols. See Table 1 for details.

The unit cell parameters of synthesised brannerite phases were determined by LeBail analysis of XRD data (Table 1); the weighted average cation radii (r_w_) were estimated using appropriate Shannon radii (assuming as-batched compositions with U present as U^5+^ only)^25^. When compared to previously reported unit cell parameters for ThTi_2_O_6_^26^, the synthesised brannerites produced in this work had smaller overall unit cell volumes, in accordance with their reduced weighted average cation radii, as summarised in Table 2 and shown in Fig. 2 (*e.g.* 228.96(1) Å^3^ for nominal composition (Th_0.80_U_0.10_Gd_0.10_)Ti_2_O_6_ with *r*_*w*_ = 0.8506 Å; compared to 231.21 Å^3^ reported for ThTi_2_O_6_ with *r*_*w*_ = 0.8567 Å). The unit cell parameters *b* and *c*, and the angle β, generally decreased with the weighted average cation radii, as shown in Fig. 2; the change in the *a*-parameter was comparably small and hence a clear trend was not apparent. These observations are consistent with previous systematic studies of the response of the brannerite crystal structure to substitution on the U/Th and/or Ti sites^13,21^.

### U L_3_ edge XANES

**Figure 3:**
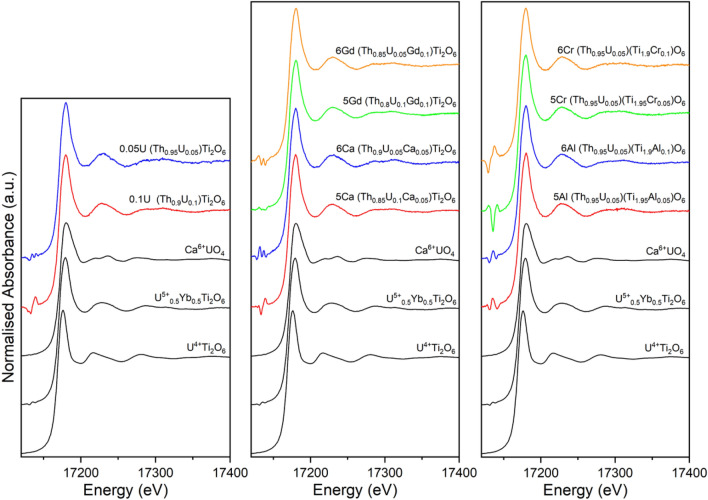
U L_3_ edge XANES spectra for ThTi_2_O_6_ compositions, adopting the brannerite structure, targeting U^5+^ and U^6+^ incorporation with appropriate charge compensation (see Table 1). The spectra of U^4+^Ti_2_O_6_, U^5+^_0.5_Yb_0.5_Ti_2_O_6_ and CaU^6+^O_4_ reference compounds are included for comparison. The corresponding average U oxidation states as-calculated by a linear regression are detailed in Table 2.

U L_3_ edge XANES data were acquired to assess the bulk U oxidation state. The position of the U L_3_ edge is dependent on the average U oxidation state, with some contribution from the local coordination environment of the U absorber. Initial examination of the acquired spectra, and U L_3_ edge positon (see Fig. 3), suggested that U^5+^ was the dominant oxidation state in all materials (edge position determined by the energy position of the maximum in the first derivative). The presence of a high concentration of Th in all compositions examined, with the Th L_3_ edge at 16,300 eV, combined with the low U concentration (U L_3_ edge at 17,166 eV), resulted in very small edge steps and incomplete normalisation of monochromator glitches in the pre-edge regions.

Linear regression of the U L_3_ edge positions of the reference compounds (U^4+^Ti_2_O_6_, U^5+^_0.5_Yb_0.5_Ti_2_O_6_ and CaU^6+^O_4_) was used to estimate the average bulk U oxidation state of the brannerite materials reported here. All estimated average U oxidation states were in the range 4.8(2) + to 5.1(2) + , as shown in Table 2. The highest U oxidation states were determined for (Th_0.85_U_0.05_Gd_0.10_)Ti_2_O_6_, with sufficient Gd^3+^ to charge balance 0.05 f.u. U^6+^ (6Gd), and (Th_0.95_U_0.05_)(Ti_1.90_Cr_0.10_)O_6_, with sufficient Cr^3+^ to charge balance 0.05 f.u. U^6+^ (6Cr), both of which had estimated U oxidation states of 5.1(2) + .

As the individual contributions of U^4+^, U^5+^ and U^6+^ cannot be deconvoluted by conventional U L_3_ edge XANES, due to the core–hole lifetime broadening of the spectra, HERFD U M_4_ edge XANES spectra were also acquired to ascertain, conclusively, the contributions of the different U oxidation states to the overall average speciation.

### HERFD U M_4_ edge XANES

**Figure 4:**
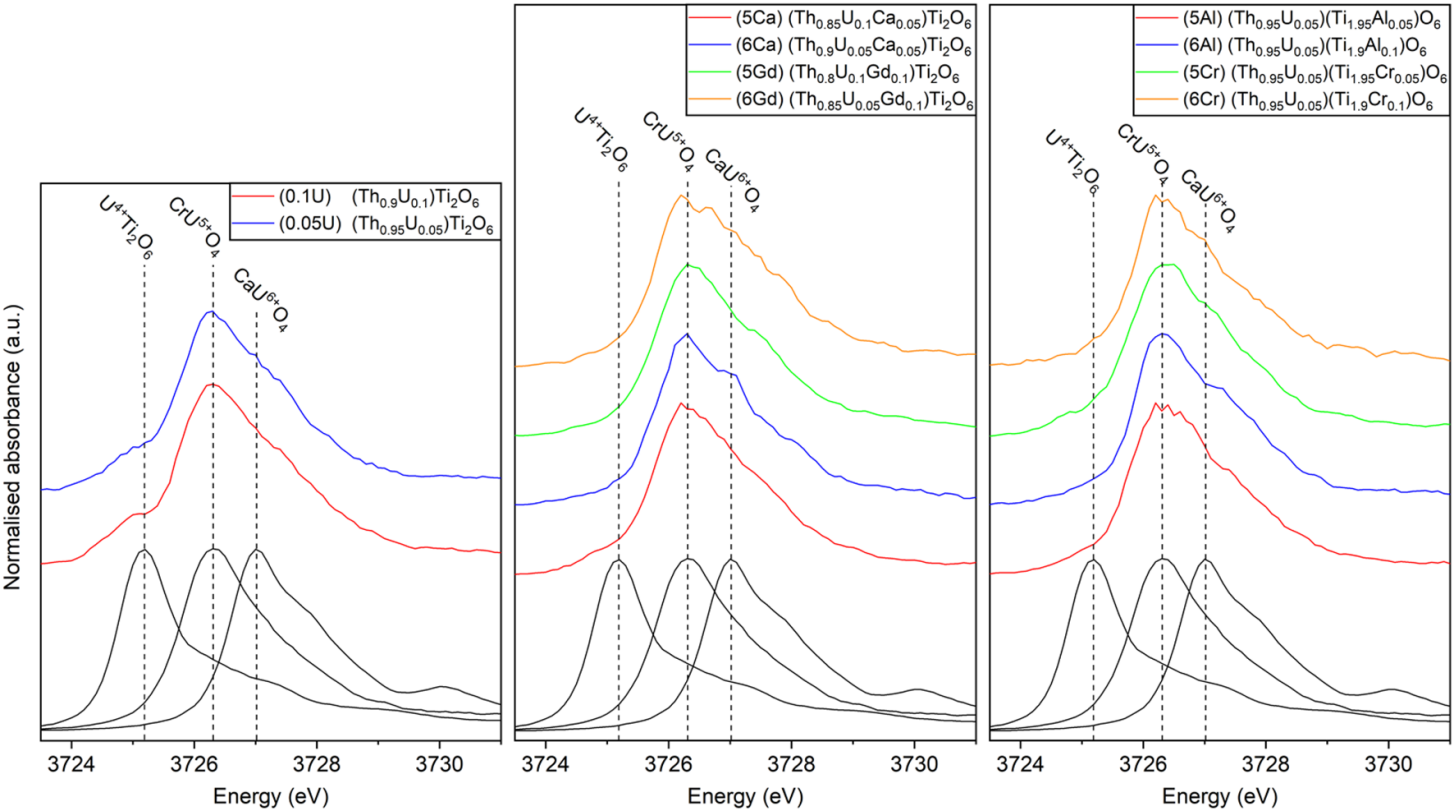
HERFD U M_4_ edge XANES of ThTi_2_O_6_ compositions, adopting the brannerite structure, targeting U^5+^ and U^6+^ incorporation with appropriate charge compensation (see Table 1). The spectra of U^4+^Ti_2_O_6_, CrU^5+^O_4_ and CaU^6+^O_4_ reference compounds are included for comparison. The average U oxidation states determined from LCF and ITFA are detailed in Table 2.

HERFD U M_4_ edge XANES allows for a more definitive determination of average U oxidation state due to greater relative separation between the edge positions of U^4+^, U^5+^ and U^6+^ and reduced core–hole lifetime broadening^28^. As with the U L_3_ edge spectra discussed above, the low U and high Th concentrations, in the materials examined, resulted in low intensities of the HERFD U M_4_ spectra. The spectra of U^4+^Ti_2_O_6_, CrU^5+^O_4_ and CaU^6+^O_4_ reference compounds were also acquired for comparison and to support further quantitative analyses. Consistent with analysis of U L_3_ edge spectra, initial examinations of the HERFD U M_4_ edge XANES supported an average oxidation state close to U^5+^, evidenced by the principle feature at *ca.* 3726 eV, as shown in Fig. 4. Inspection of the HERFD U M_4_ edge XANES of (Th_0.90_U_0.10_)Ti_2_O_6_ and (Th_0.95_U_0.05_)Ti_2_O_6_, also identified a small but distinct contribution at *ca.* 3725 eV, Fig. 4, demonstrating the presence of an additional minor component of U^4+^. Inspection of the HERFD U M_4_ edge XANES of some compositions targeting U^6+^, most obviously (Th_0.90_U_0.05_Ca_0.05_)Ti_2_O_6_ and (Th_0.95_U_0.05_)(Ti_1.90_Al_0.10_)O_6_, evidenced a small contribution at *ca.* 3727.5 eV, Fig. 4, attributed to the presence of U^6+^^28^. The HERFD U M_4_ edge XANES of materials with a uranyl speciation exhibit additional post-edge features, observed at *ca.* 3730 eV for CaU^6+^O_4_, see Fig. 4. ^28,29^ These features were not observed in any of the HERFD U M_4_ spectra of the materials studied here, demonstrating that, although detectable fractions of U^6+^ were present (see Table 2), uranate, rather than uranyl, is the dominant U^6+^ speciation. Our confidence in this statement is tempered by the signal to noise ratio of the data and, certainly, a minor fraction of uranyl speciation cannot be conclusively ruled out.

Linear combination fitting (LCF) was utilised to estimate the proportions of U^4+^, U^5+^, and U^6+^ and the average U oxidation state. Consistent with analysis of U L_3_ edge spectra, LCF of the HERFD U M_4_ edge spectra evidenced average U^5+^ speciation, within the range 4.91(12) + to 5.12(12) + (detailed in Table 2). The compositions (Th_0.90_U_0.10_)Ti_2_O_6_ and (Th_0.95_U_0.05_)Ti_2_O_6_ were the least oxidised, with average U oxidation states of 4.93(12) + and 4.91(12) +, and U^4+^ fractions of 14.9(20)% and 13.3(32)%, respectively. The most oxidised materials were: (Th_0.85_U_0.05_Gd_0.10_)Ti_2_O_6_ and (Th_0.95_U_0.05_)(Ti_1.90_Al_0.10_)O_6_, with average U oxidation states of 5.12(11) + and 5.10(13) +, respectively. Fitting of the spectra of these compositions also necessitated a small but significant contribution from the U^6+^ reference compound (CaU^6+^O_4_), of 14.6(11)% and 10.2(14)% respectively. These observations are in keeping with the oxidation states estimated from the U L_3_ edge spectra, as well as previous reports of a fraction of retained U^4+^ in air-synthesised charge compensated brannerites^10,21^.

Iterative Target Transformation Factor Analysis (ITFA) was also performed to evaluate the individual contributions of U^4+^, U^5+^ and U^6+^ to the final spectra and average U oxidation state. Initial principal component analysis of the brannerite and reference compounds determined that only three spectral-like components were necessary to reproduce all experimental spectra (see Supplementary Information, Fig. S1). This suggested that the spectra of the materials studied here can be well described by the three reference compounds.

Following the principal component analysis, the ITFA procedure was continued, with each of the three theoretical components accurately describing one of the reference compounds (see Supplementary Information, Fig. S1). As such, each component was assigned a given U oxidation state, with the relative fractions of each component utilised to calculate an average U oxidation state for the materials under examination. The relative fractions of the synthetic spectra and the calculated U oxidation states were both in excellent agreement with those calculated from linear combination fitting of the spectra (see Table 2; (see Supplementary Information, Figs. S2–6).

This analysis was confirmed by repeating the ITFA procedure with a larger suite of U^4+^, U^5+^ and U^6+^ reference compounds, as well as only U^4+^Ti_2_O_6_ and CaU^6+^O_4_ reference compounds, to ensure the synthetic U^5+^ spectrum was representative of the U^5+^ contribution in these materials. In both cases the relative fractions of the synthetic spectra and overall U oxidation states were in very close agreement with both the linear combination fitting and the three reference compound ITFA.

The average U oxidation states determined by all methods (linear regression of the U L_3_ edge position, and LCF and ITFA of the U M_4_ edge) are in excellent agreement (see Table 2). The material shown to have the highest U oxidation state, (Th_0.85_U_0.05_Gd_0.10_)Ti_2_O_6_, contained 13.3(1)% U^6+^ as determined by ITFA of the HERFD U M_4_ edge spectrum (detailed in Supplementary Information Table S1). Assuming target stoichiometry, this corresponds to only 0.0133 f.u. of U^6+^. No features relating to the presence of uranyl speciation were observed in the spectra of any of the materials produced here.

### Raman spectroscopy

**Figure 5:**
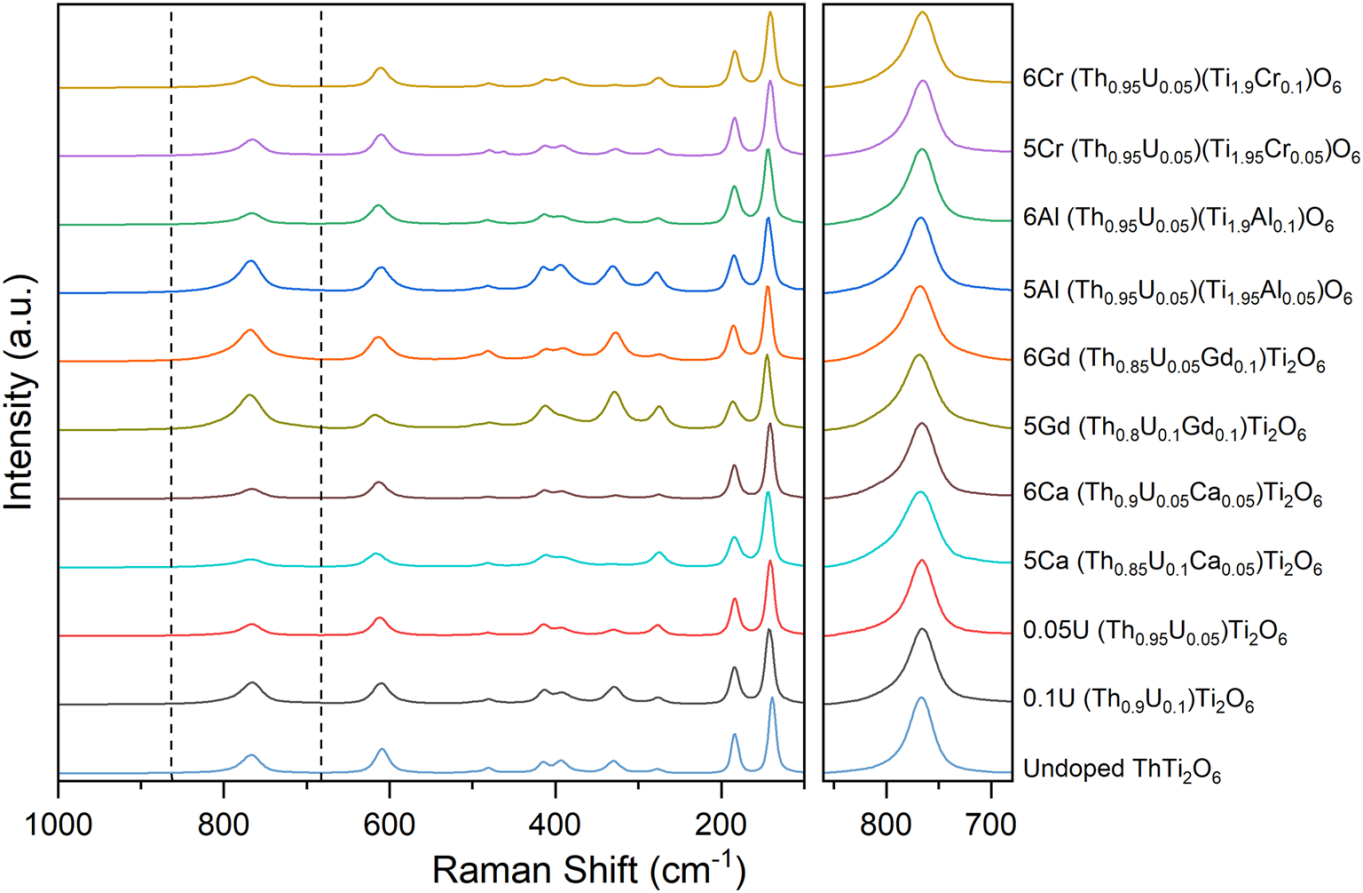
Normalised Raman spectra of ThTi_2_O_6_ compositions, adopting the brannerite structure, targeting U^5+^ and U^6+^ incorporation with appropriate charge compensation (see Table 1). The panel shows of the region from 680 to 860 cm^−1^, showing a weak and broad band centred at 780 cm^−1^ as an apparent as a shoulder on the comparatively intense and sharp band centred at 765 cm^−1^; the latter can confidently be assigned as the A_g_ symmetric stretch of TiO_6_ octahedra (spectra are normalized normalised with respect to the intensity of the A_g_ mode for comparison).

As expected from the near single phase nature of the materials produced here, determined by XRD, the Raman spectra of the brannerite compounds, Fig. 5, are in excellent agreement with previously reported spectra of actinide brannerites^15,16^. An earlier investigation of (Th_0.85_U_0.10_Ca_0.05_)Ti_2_O_6_ and (Th_0.90_U_0.05_Ca_0.05_)Ti_2_O_6_ (*i.e.* the same nominal compositions as reported here) determined the presence of uranyl species, from analysis of Raman spectra. This assessment was made by assignment of the ν_*1*_ symmetric stretch, apparent as a weak and broad band in the range 780–820 cm^−1^^15^, deconvoluted from the comparatively strong and intense band at 765–770 cm^−1^ attributed to the A_g_ symmetric stretch of the TiO_6_ octahedra. The reported ν_*1*_ stretch modes are within the wavenumber range previously determined for a diverse suite of uranyl compounds^30,31^. The ThTi_2_O_6_ brannerite compounds investigated here all exhibited evidence of a weak and broad band, centred at 780 cm^−1^, apparent as a shoulder on the comparatively intense and sharp band, centred at 765 cm^−1^, which can confidently be assigned as the A_g_ symmetric stretch of TiO_6_ octahedra. However, we also observed the Raman spectrum of ThTi_2_O_6_ to present such a shoulder on the A_g_ symmetric stretch of TiO_6_ octahedra at 765 cm^−1^, in agreement with earlier investigation^15^. It is evident that this band, also observed in the spectrum of ThTi_2_O_6_, must have at least some contribution from other Raman active modes of the brannerite structure (potentially arising from distortion of the TiO_6_ polyhedra). We believe caution should be exercised in attributing uranyl speciation in brannerite compounds by assignment of the weak and broad band deconvoluted at 780–820 cm^−1^ as the ν_*1*_ symmetric stretch. This is further evidenced by comparison of the Raman spectra of (Th_0.85_U_0.05_Gd_0.10_)Ti_2_O_6_ and (Th_0.80_U_0.10_Gd_0.10_)Ti_2_O_6_. These compositions were determined to incorporate 13.3(1)% and 6.8(1)% of U^6+^ respectively (averaged from Table 1), but the weak and broad band centred at 780 cm^−1^ is not strongly modulated, which is not consistent with a dominant uranyl speciation. We consider Raman spectroscopy to be inconclusive with regard to determination of uranyl speciation in the brannerite compounds reported here. If present, the concentration of uranyl species in the compounds examined in this work are very low, with even the most oxidised material containing only 0.0133 f.u. of U^6+^ as noted above. It should also be noted that as a result of the short excitation wavelengths utilised in standard Raman spectroscopy, it is a potentially surface sensitive technique with observations made from spectra not necessarily being representative of the bulk material, particularly in materials highly absorbing in the excitation laser regimes (532 nm) relevant to this work.

## Discussion

The aim of this study was to further investigate the stability of U^6+^ in the brannerite structure, in four ThTi_2_O_6_ solid solutions, targeting U^5+^ or U^6+^ speciation, stabilised by co-substitution and charge compensation on the Th or Ti site. The materials studied here were produced to examine the possibility of stabilising a significant fraction of U^6+^ within the brannerite structure. HERFD U M_4_ edge spectra provided direct and unambiguous evidence for a small fraction of U^6+^ in all materials, however, U^5+^ was the major U oxidation state present (exceeding 77%, from ITFA). U^5+^ was dominant whether sufficient charge balancing species were present to permit oxidation of all U present to U^6+^ or not, or indeed when no charge balancing species were present (*e.g.* (Th_0.90_U_0.10_)Ti_2_O_6_, 15.7(1)% U^4+^, 77.3(6)% U^5+^, 6.9(1)% U^6+^, from ITFA). However, it is interesting to note that the average U oxidation states of materials targeting U^6+^ were generally characterised by a greater fraction of U^6+^ speciation, compared to those targeting U^5+^ speciation; for example, (Th_0.85_U_0.05_Gd_0.10_)Ti_2_O_6_ and (Th_0.80_U_0.10_Gd_0.10_)Ti_2_O_6_, which were determined to incorporate 13.3(1)% and 6.8(1)% U^6+^ respectively from ITFA. Compositions designed to target U^6+^ speciation were determined to have a lower average oxidation sate, closer to U^5+^, which is likely to be realised by a low concentration of cation and/or oxygen vacancies, consistent with the detection of trace ThO_2_ and TiO_2_ impurities, and the known defect chemistry of the brannerite structure^27,32,33^. An earlier XPS study of (Th_0.55_U_0.30_Ca_0.15_)Ti_2_O_6_, with sufficient Ca^2+^ to charge balance U^5+^, evidenced a higher contribution of U^4+^ to the U 4f_7/2_ peak when collected on a polished surface, compared to an unpolished fracture surface^10^. This was attributed to apparent Ca^2+^ and U^5+^ and/or U^6+^ enrichment at the grain boundaries. Such enrichment of charge compensating elements in the grain boundaries of the materials studied here could also result in the determined bulk average oxidation state being lower than targeted, in addition to any cation and/or anion vacancies.

U L_3_ edge and HERFD U M_4_ edge XANES are effectively bulk techniques, giving insight into the average U oxidation states and environments throughout a material. HERFD U M_4_ edge spectra provided direct and unambiguous evidence for a small fraction of U^6+^ in all materials, however, no features characteristic of uranyl speciation were observed, even in the most oxidised material, (Th_0.85_U_0.05_Gd_0.10_)Ti_2_O_6_, with 13.3(1)% U^6+^ from ITFA. Although Raman spectra evidenced a weak and broad band centred at 780 cm^−1^, potentially characteristic of a ν_*1*_ symmetric stretch of uranyl species^15,16^, the evident presence of this band in the spectrum of ThTi_2_O_6_ means that caution must be exercised in its diagnostic attribution to the presence of uranyl species in the brannerite structure. In comparison with X-ray spectroscopies, Raman spectroscopy using a 532 nm laser has a relatively low penetration depth in polycrystalline opaque materials such as those examined in this work, and may be more sensitive to uranyl speciation formed by surface oxidation. The data presented here directly and conclusively demonstrate the stabilisation of a small fraction of U^6+^ in the brannerite structure, but the balance of evidence tends to support uranate, rather than uranyl, as the dominant U^6+^ bulk speciation. Raman spectroscopy has proven more conclusive in substantiating the presence of uranyl speciation in metamict mineral brannerite, however, this may reflect past aqueous alteration of the specimen consistent with the observation of U-OH bending vibrations^34^.

A wide range of uranium (VI) oxometallates may be synthesised at high temperature in either air or oxygen atmosphere, so the partial pressure of oxygen is not thought to be the limiting factor in stabilisation of only a modest U^6+^ concentration in the ThTi_2_O_6_ compounds reported here^35^. The unit cell volume and weighted average cation radius of these compounds were shown to be well within the actinide brannerite stability field, as shown by Fig. 2, which suggests that ionic size effects are also unlikely to be limiting of the U^6+^ concentration. The brannerite structure comprises corrugated sheets of BO_6_ octahedra, in which each BO_6_ octahedron shares three edges, with the sheets connected by chains of edge sharing AO_6_ octahedra, in which each AO_6_ octahedron shares two edges. In ThTi_2_O_6_, across the shared octahedral edges, the A…A cation distance is 3.823 Å, and the B…B cation distances are 3.039 and 3.161 Å^22^. Substitution of U^6+^ at either site would be expected to give rise to highly unfavourable electrostatic repulsions, as a consequence of the short approach distance to neighbouring cations, which we consider may be limiting on the concentration of U^6+^ incorporation.

## Conclusions

ThTi_2_O_6_ compounds with the brannerite structure were designed to incorporate U^5+^ or U^6+^, at dilute concentration of 0.10 or 0.05 formula units, by appropriate charge compensation involving co-substitution of Gd^3+^, Ca^2+^, Al^3+^, or Cr^3+^. Near-single-phase compounds were produced in each case. X-ray absorption spectroscopies evidenced a majority U^5+^ speciation in all compounds, regardless of the nature and relative fraction of the charge compensating species. All compositions exhibited a small contribution from a U^6+^ oxidation state, as evidenced from HERFD U M_4_ edge XANES, and in many cases also a small contribution of U^4+^. Compositions targeting only U^6+^ were, in general, determined to contain a greater contribution of U^6+^. Although Raman spectra presented a weak and broad band plausibly indicative of uranyl speciation, the observation of this band from ThTi_2_O_6_, means that caution must be exercised in its diagnostic attribution to the presence of uranyl species in the brannerite structure. In contrast, no spectroscopic signatures of uranyl speciation were apparent in the HERFD U M_4_ edge XANES, although the signal to noise ratio of the data presented here mean that a minor contribution of uranyl speciation cannot be ruled out. Therefore, the balance of our evidence tends to support uranate rather than uranyl, as the dominant U^6+^ speciation in the brannerites reported here.

This investigation has demonstrated that the ThTi_2_O_6_ brannerite structure can incorporate a small fraction of U^6+^, alongside a more significant inventory U^5+^, by charge compensation on the Th or Ti site. Stabilisation of U^6+^ was not thought to be limited by the weighted average cation radii, which were within the stability field of the actinide brannerites, or conditions of synthesis, which were conducive to formation of U^6+^ oxometallates. It is considered that the U^6+^ concentration may be limited by unfavourable electrostatic repulsion arising from substitution in the octahedral Th or Ti sites, which share two or three edges, respectively, with neighbouring polyhedra in the brannerite structure.

## Methods

Materials were prepared by solid state reaction and sintering. Stoichiometric amounts (see Table 2) of UO_2_, ThO_2_ (produced by decomposition of Th(NO_3_)_4_·5H_2_O at 550 °C), TiO_2_ (anatase), CaTiO_3_, Gd_2_O_3_, Al_2_O_3_, and Cr_2_O_3_ were homogenised by high energy ball milling (Fritsch Pulverisette 23 reciprocating ball mill, 30 Hz, 5 min) utilising yttria-stabilised zirconia mill pots and milling media, with isopropanol as a carrier fluid. The milled slurries were dried at 85 °C, and the resulting powder cakes broken up by hand in a mortar and pestle. The milled powders were then pressed into 10 mm pellets under 2 t (approx. 250 MPa). Pellets were heat treated in alumina crucibles at 1400 °C for 24 h in air.

X-ray diffraction (XRD) patterns of each sample were collected on powdered material (Bruker D2 Phaser, Ni-filtered Cu K_α_ radiation). Phase analysis was conducted by matching the reflections observed to phases in the PDF-4 + database^36^. Unit cell parameters of the brannerite phase in each composition were derived using LeBail method refinements, utilising the Topas^37^ and JEdit^38^ software packages. The background of each diffraction pattern was modelled with an eight term shifted Chebyshev polynomial; peak shapes resulting from instrumental and sample-based contributions were modelled using modified Thompson-Cox-Hastings (TCHZ) pseudo-Voigt functions.

Scanning electron microscopy with coupled energy dispersive X-ray spectroscopy (SEM/EDX) was used to confirm incorporation of the dopant cations. Due to the significant overlap between the Th M_β_ and U M_α,1_ emissions, accurate compositional analysis was not possible. Solid samples were prepared for SEM/EDX analysis by mounting in a cold-set epoxy resin, polishing with increasingly fine grades of diamond suspensions, before coating with a conductive carbon layer. Backscattered electron micrographs and EDX spectra were collected using a Hitachi TM3030 (operating at 15 kV) microscope with Bruker Quantax 70 EDX system.

Raman spectra were collected on polished materials prior to carbon coating. Spectra at various points across the polished surfaces were collected using a Horiba XploRa PLUS Raman microscope with a 532 nm laser at 100 × magnification. The instrument was calibrated using the 520.7 cm^−1^ band of a Si wafer.

U L_3_ edge XANES were acquired at Diamond Light Source (DLS) beamline B18. The incident energy was selected using a Si (111) monochromator, with data collected in transmission mode from 16,970 to 17,560 eV. The intensities of the incident and transmitted beams were measured using ionisation chambers, operating in a stable region of their I/V curve. The K edge of an Y foil was used to calibrate the spectra, with the first peak in the first derivative of the Y foil spectrum set to 17,038 eV. The spectra of well-characterised specimens of UTi_2_O_6_ (U^4+^, 6 coordinate), U_0.5_Yb_0.5_Ti_2_O_6_ (U^5+^, 6 coordinate), and CaUO_4_ (U^6+^, 8 coordinate) were collected to act as reference compounds of known U oxidation state. The energy position, E_0_, of each spectrum was determined by the energy position of the maximum in the first derivative.

High Energy Resolution Fluorescence Detected (HERFD) U M_4_ edge XANES were collected at ESRF beamline BM20^39^. The incident energy was selected using the (111) reflection from a double Si crystal monochromator. XANES spectra were measured in HERFD mode using an X-ray emission spectrometer^40^. The sample, analyser crystal, and photon detector (Si drift detector) were arranged in a vertical Rowland geometry. HERFD U M_4_ edge spectra were obtained by recording the maximum intensity of the U M_β_ emission line (*ca.* 3337 eV) as a function of the incident energy. The emission energy was selected using the (220) reflection of five spherically bent striped Si crystal analysers (1 m bending radius) aligned at a 75° Bragg angle. The paths of the incident and emitted X-rays were minimised, and the sample was maintained under He atmosphere, in order to avoid losses in intensity due to absorption. Spectra of well-characterised specimens of UTi_2_O_6_, CrUO_4_, and CaUO_4_ were also acquired to act as reference compounds of known U oxidation state (U^4+^, U^5+^, and U^6+^ respectively).

Both U L_3_ and HERFD U M_4_ edge spectra were processed, and linear combination fitting of the U M_4_ spectra performed, in Athena, part of the Demeter software package^41,42^. For Iterative Target Transformation Factor Analysis (ITFA), data were normalised using PyMca^43^ and were analysed using the ITFA software package^44,45^, to further define the oxidation state of U within the samples^20,46–48^. Recent work has suggested that the errors on this analysis have not been fully quantified^49,50^, and therefore these values are considered to have a margin of error of 5%. Principal component analysis showed three components were generally necessary to describe the HERFD U M_4_ edge spectra (those of the materials studied here plus U^4+^Ti_2_O_6_, Cr^5+^UO_4_ and CaU^6+^O_4_ reference compounds), and the initial fractions of the three components were set to unity for one of each of the reference compounds. The iterative target test procedure was then utilised to find the relative concentrations of the three components in the spectra of the materials studied here, with each component representing U^4+^, U^5+^ or U^6+^ according to comparisons with the reference compounds used. For validation, the procedure was then repeated twice, once including only U^4+^Ti_2_O_6_, CrU^5+^O_4_ and CaU^6+^O_4_ reference compounds and once including a wider range of reference compounds (CaU^6+^O_4_, SrU^6+^O_4_, U^6+^TiO_5_, CrU^5+^O_4_, U^5+^MoO_5_, U^4+^O_2_ and U^4+^Ti_2_O_6_). In both cases principal component analysis showed that three components were necessary to describe the spectra.

### Supplementary Information

Supplementary Information.

## Data Availability

The datasets generated during and/or analysed during the current study are available from the corresponding author on reasonable request.
