# Supplementary material for: A multimodal X-ray spectroscopy investigation of uranium speciation in ThTi2O6 compounds with the brannerite structure
Source: Sci Rep. 2023 Aug 7;13:12776. doi: 10.1038/s41598-023-38912-1 (PMC10406819; doi:10.1038/s41598-023-38912-1)
Supplement: Supplementary file 1 — Supplementary Information. [file 41598_2023_38912_MOESM1_ESM.docx]

Supplementary Information:

A multimodal X-ray spectroscopy investigation of uranium speciation in ThTi_2_O_6_ compounds with the brannerite structure

Malin C. Dixon Wilkins^1,2^, Luke T. Townsend^1^, Martin C. Stennett^1^, Kristina O. Kvashnina^3,4^, Claire L. Corkhill^1,5*^, Neil C. Hyatt^1,2,5^

^1^Department of Materials Science and Engineering, University of Sheffield, Sheffield, UK

^2^School of Mechanical and Materials Engineering, Washington State University, Pullman, WA, 99164, USA

^3^The Rossendorf Beamline at ESRF, CS 40220, 38043 Grenoble Cedex 9, France

^4^Helmholtz-Zentrum Dresden-Rossendorf, Institute of Resource Ecology, Bautzner Landstrasse 400, 01328 Dresden, Germany.

^5^School of Earth Sciences, The University of Bristol, Bristol, BS8 1RL, UK.

*Corresponding author: [c.corkhill@sheffield.ac.uk](mailto:c.corkhill@sheffield.ac.uk)


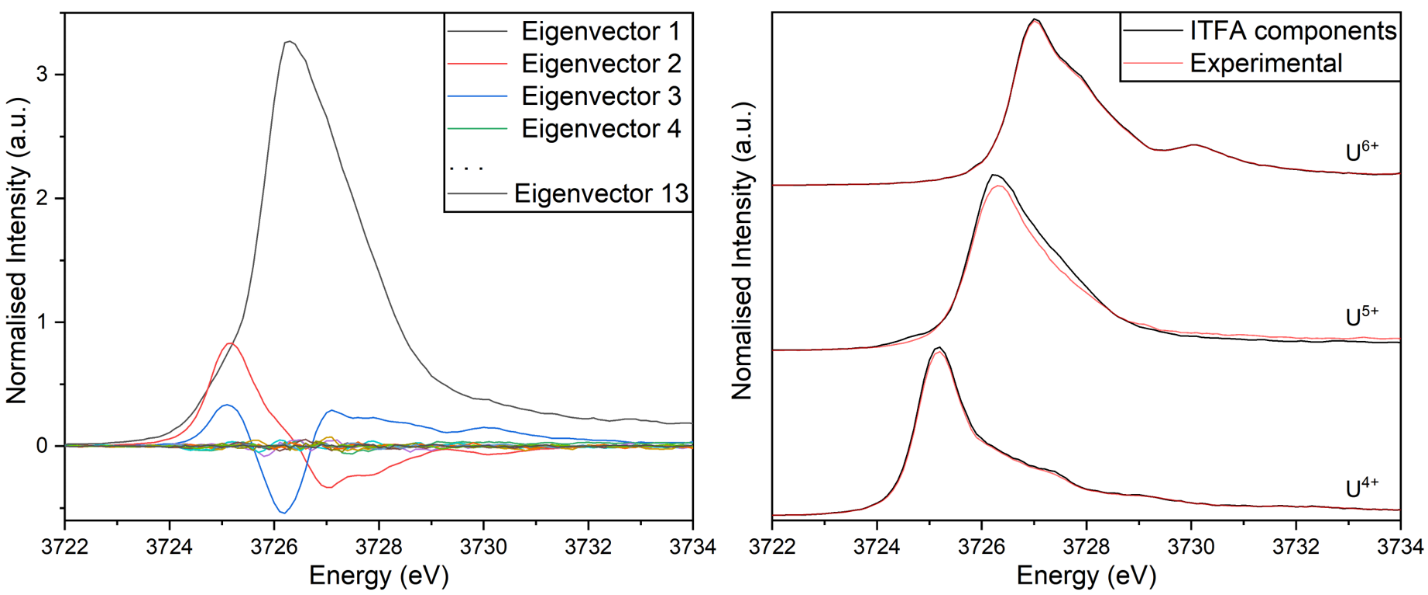


Figure S1: Plots of the ITFA extracted eigenvectors, showing spectral-like behavior for eigenvectors 1 – 3 only (left). ITFA derived isolated single component spectra relating to the contributions of different U oxidation states (black), overlaid with experimental HERFD U M_4_ edge spectra of U^4+^Ti_2_O_6_, CrU^5+^O_4_, and CaU^6+^O_4_ (right).


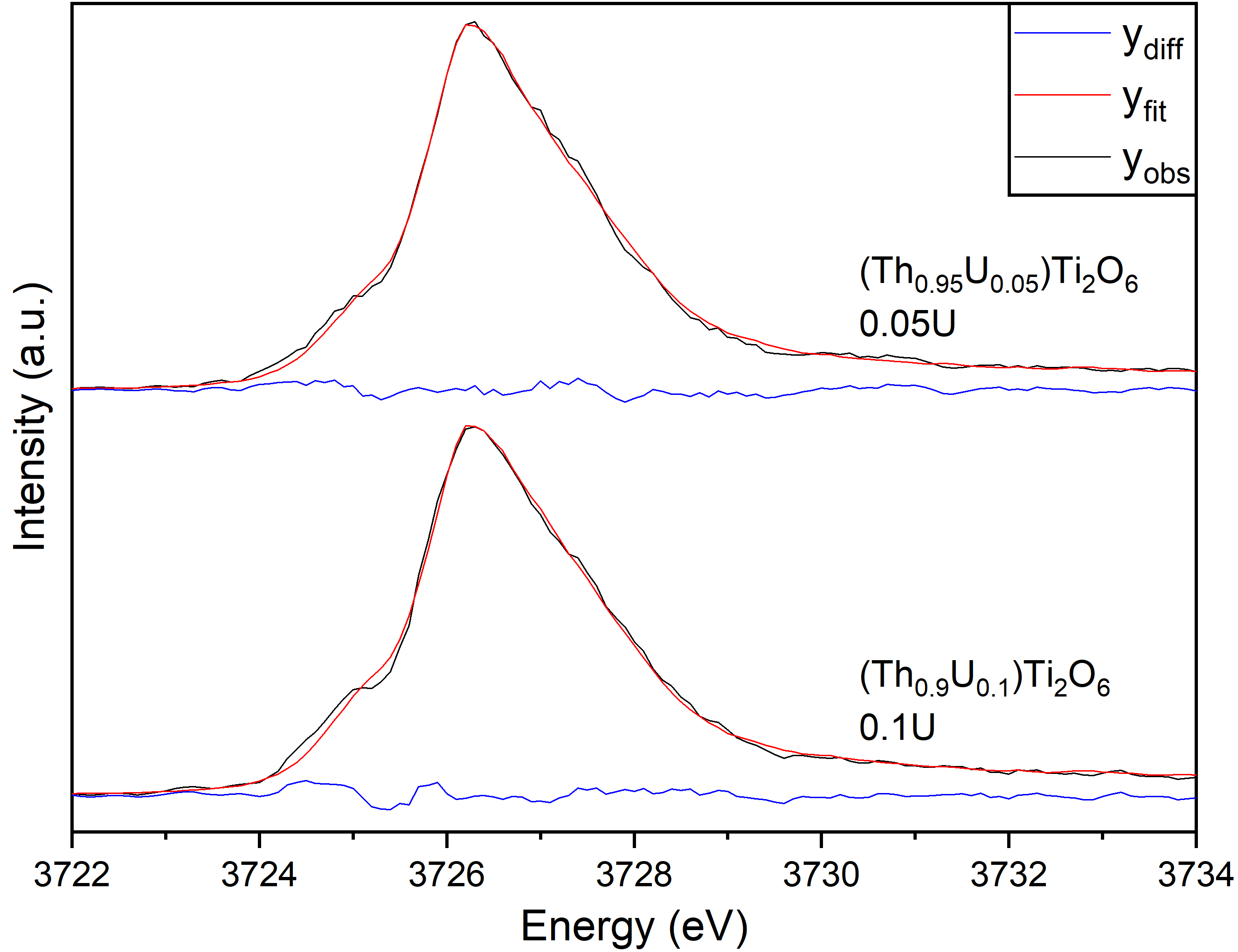


Figure S2: Experimental (black), ITFA reproduced (red) and difference (blue) HERFD U M_4_ edge spectra of (Th_0.90_U_0.10_)Ti_2_O_6_ (0.1U) and (Th_0.95_U_0.05_)Ti_2_O_6_ (0.05U).


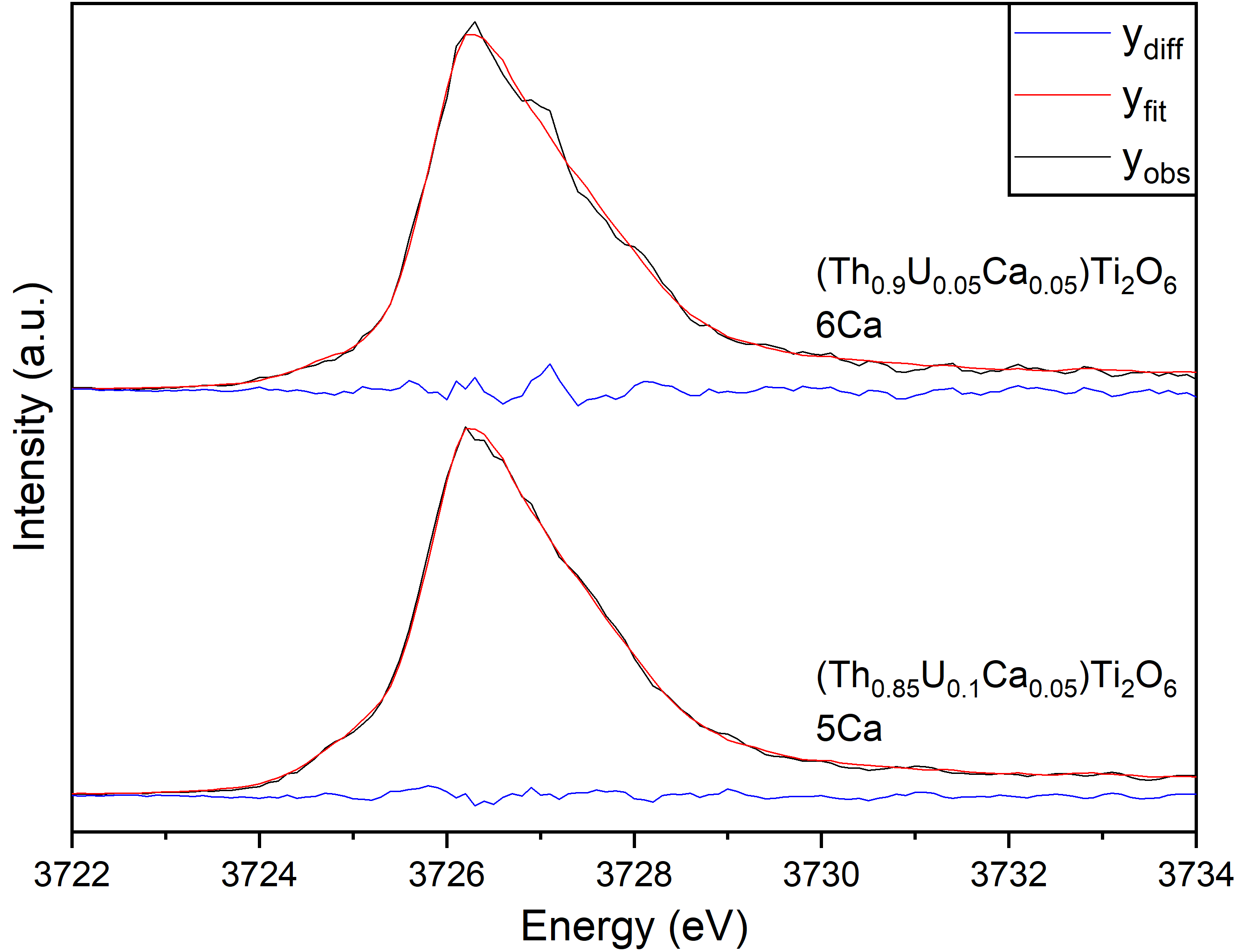


Figure S3: Experimental (black), ITFA reproduced (red) and difference (blue) HERFD U M_4_ edge spectra of (Th_0.85_U_0.10_Ca_0.05_)Ti_2_O_6_ (5Ca) and (Th_0.90_U_0.05_Ca_0.05_)Ti_2_O_6_ (6Ca).


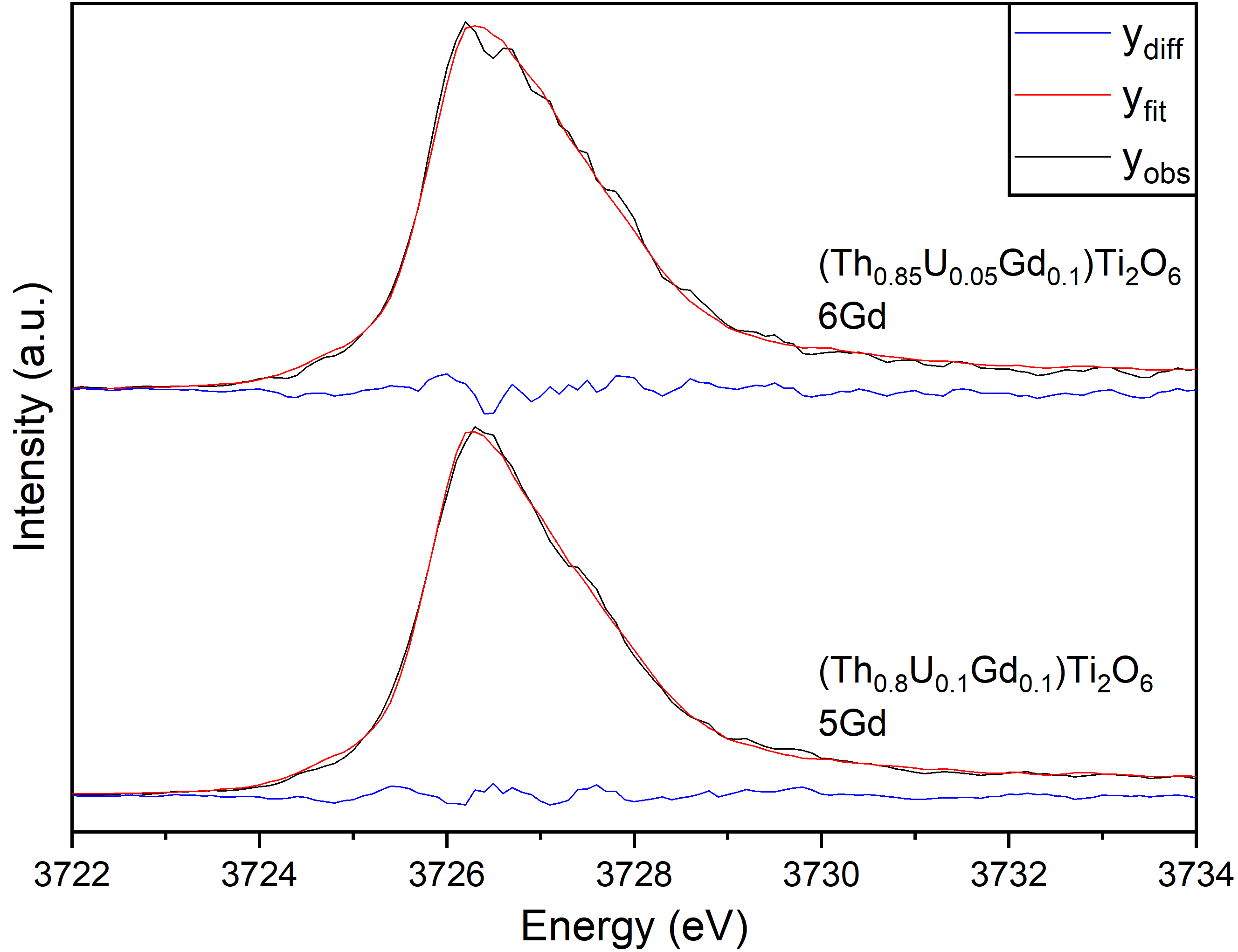


Figure S4: Experimental (black), ITFA reproduced (red) and difference (blue) HERFD U M_4_ edge spectra of (Th_0.80_U_0.10_Gd_0.10_)Ti_2_O_6_ (5Gd) and (Th_0.85_U_0.05_Gd_0.10_)Ti_2_O_6_ (6Gd).


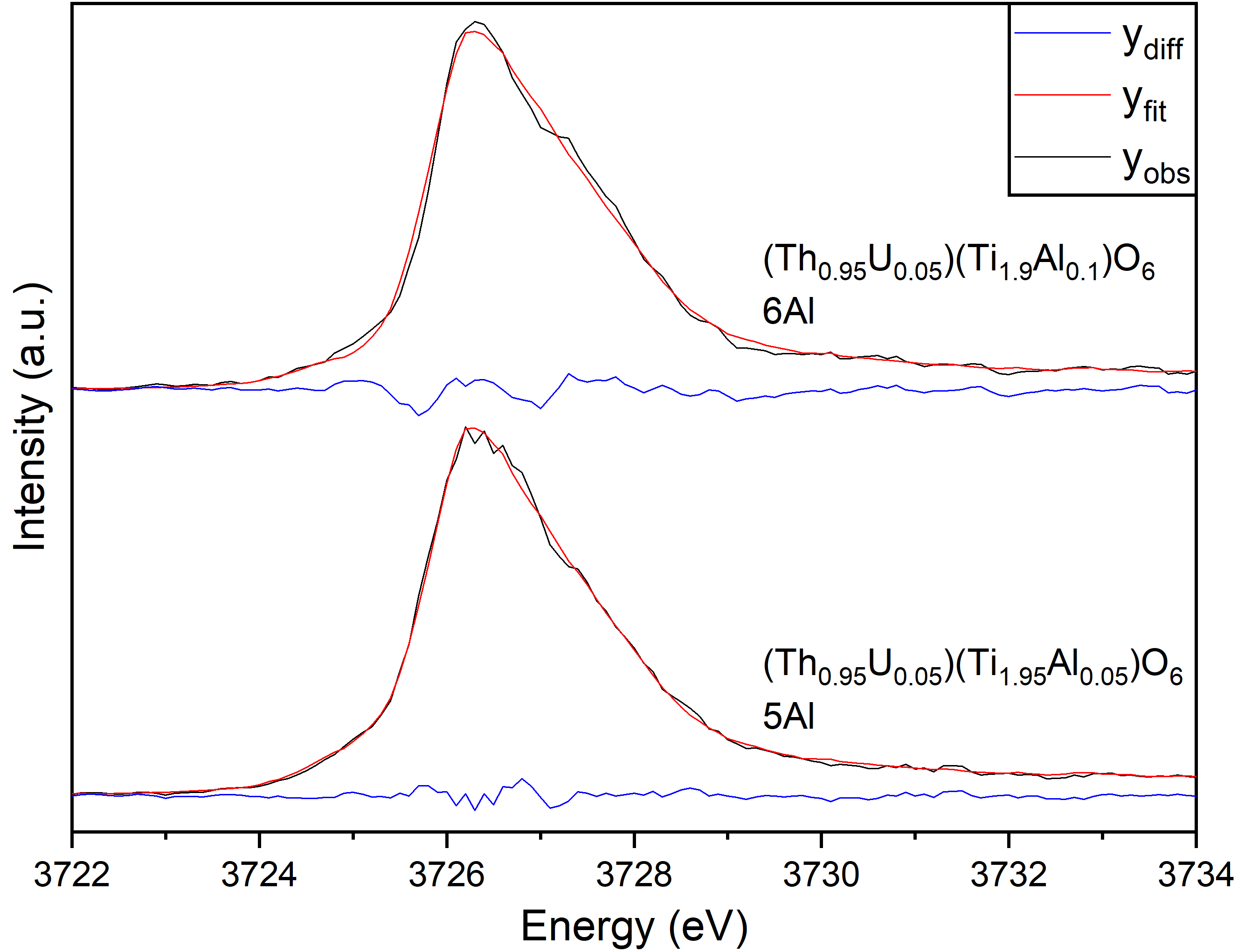


Figure S5: Experimental (black), ITFA reproduced (red) and difference (blue) HERFD U M_4_ edge spectra of (Th_0.95_U_0.05_)(Ti_1.95_Al_0.05_)O_6_ (5Al) and (Th_0.95_U_0.05_)(Ti_1.90_Al_0.10_)O_6_ (6Al).


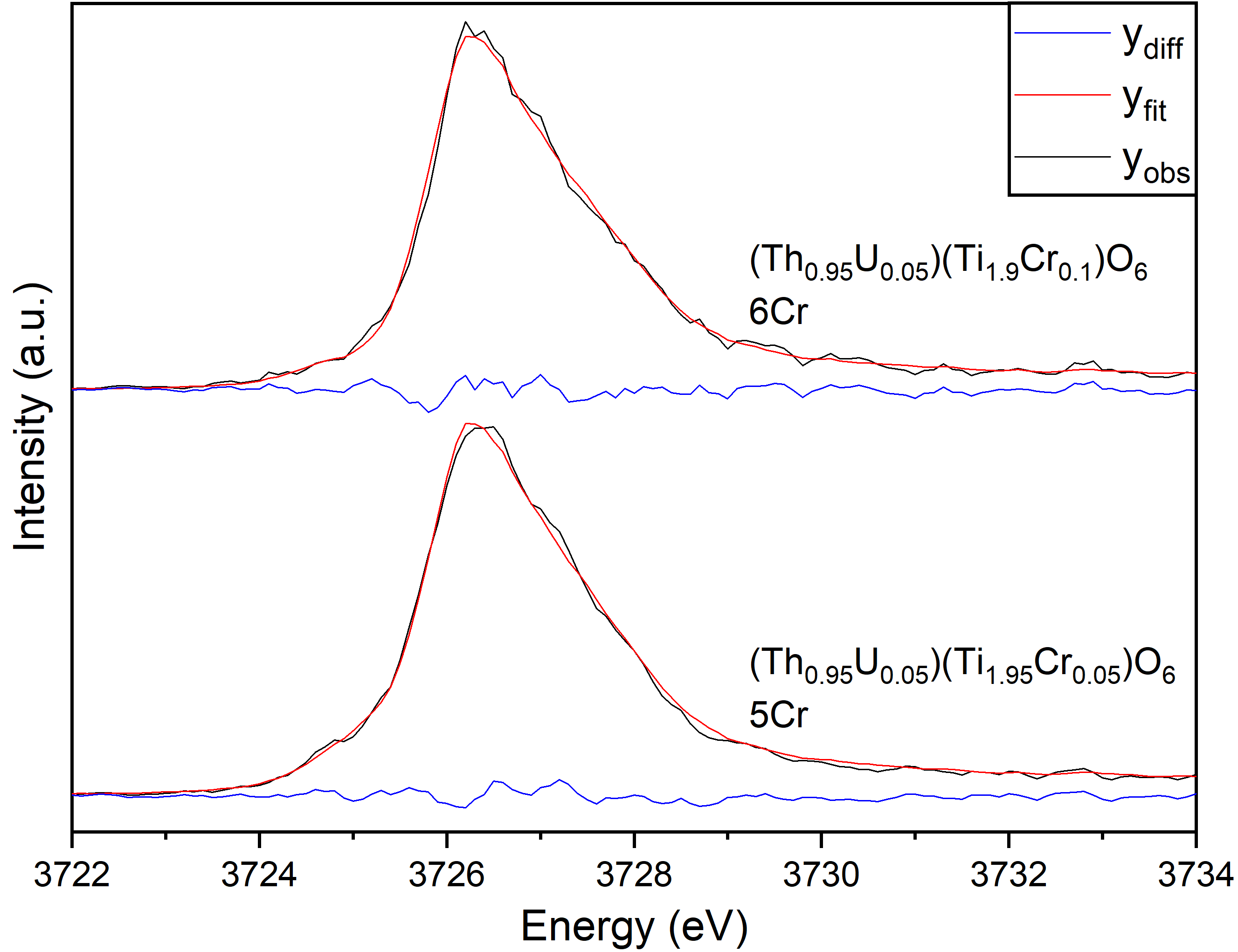


Figure S6: Experimental (black), ITFA reproduced (red) and difference (blue) HERFD U M_4_ edge spectra of (Th_0.95_U_0.05_)(Ti_1.95_Cr_0.05_)Ti_2_O_6_ (5Cr) and (Th_0.95_U_0.05_)(Ti_1.90_Cr_0.10_)O_6_ (6Cr).

Table S1: Results from Iterative Target Transformation Analysis (ITFA) and Linear Combination Fitting (LCF) of the HERFD U M_4_ edge XANES spectra of ThTi_2_O_6_ compositions, adopting the brannerite structure, targeting U^5+^ and U^6+^ incorporation with appropriate charge compensation (see Table 1). ITFA was performed using three components as determined by principal components analysis.

|  |  |  |  | ITFA |  |  |  |  | LCF |  |  |
| --- | --- | --- | --- | --- | --- | --- | --- | --- | --- | --- | --- |
| Nominal Composition | ID | U ox. state | U^4+^ (%) | U^5+^ (%) | U^6+^ (%) | R factor | U ox. state | U^4+^Ti_2_O_6_ (%) | CrU^5+^O_4_ (%) | CaU^6+^O_4_ (%) | R factor |
| (Th_0.90_U_0.10_)Ti_2_O_6_ | 0.1U | 4.91(6) | 15.7(1) | 77.3(6) | 6.9(1) | 0.00138 | 4.93(12) | 14.9(20) | 76.8(13) | 8.3(11) | 0.00795 |
| (Th_0.95_U_0.05_)Ti_2_O_6_ | 0.05U | 4.89(6) | 14.2(1) | 82.5(6) | 3.4(1) | 0.00111 | 4.91(12) | 13.3(32) | 81.9(13) | 4.8(11) | 0.00795 |
| (Th_0.85_U_0.10_Ca_0.05_)Ti_2_O_6_ | 5Ca | 4.96(6) | 7.7(1) | 89.0(7) | 3.3(1) | 0.00059 | 4.98(9) | 7.1(13) | 87.9(10) | 5.1(9) | 0.00437 |
| (Th_0.90_U_0.05_Ca_0.05_)Ti_2_O_6_ | 6Ca | 5.04(6) | 1.1(1) | 93.6(7) | 5.3(1) | 0.00185 | 5.06(9) | 0.8(1) | 92.0(12) | 7.2(10) | 0.00524 |
| (Th_0.80_U_0.10_Gd_0.10_)Ti_2_O_6_ | 5Gd | 5.04(6) | 2.7(1) | 90.5(7) | 6.8(1) | 0.00097 | 5.06(7) | 2.2(2) | 89.5(8) | 8.3(7) | 0.00280 |
| (Th_0.85_U_0.05_Gd_0.10_)Ti_2_O_6_ | 6Gd | 5.11(6) | 2.6(1) | 84.1(6) | 13.3(1) | 0.00194 | 5.12(11) | 2.3(2) | 83.1(12) | 14.6(11) | 0.00646 |
| (Th_0.95_U_0.05_)(Ti_1.95_Al_0.05_)O_6_ | 5Al | 5.02(6) | 3.8(1) | 90.2(7) | 6.0(1) | 0.00099 | 5.04(9) | 3.3(4) | 89.1(11) | 7.5(10) | 0.00472 |
| (Th_0.95_U_0.05_)(Ti_1.9_Al_0.10_)O_6_ | 6Al | 5.09(6) | 0 | 91.2(7) | 8.8(1) | 0.00242 | 5.10(13) | 0 | 89.8(16) | 10.2(14) | 0.01015 |
| (Th_0.95_U_0.05_)(Ti_1.95_Cr_0.05_)O_6_ | 5Cr | 4.98(6) | 6.8(1) | 88.7(7) | 4.5(1) | 0.00133 | 5.00(11) | 6.3(11) | 87.3(13) | 6.4(11) | 0.00630 |
| (Th_0.95_U_0.05_)(Ti_1.9_Cr_0.10_)O_6_ | 6Cr | 5.02(6) | 0 | 97.6(7) | 2.4(1) | 0.00212 | 5.04(10) | 0 | 95.8(13) | 4.2(11) | 0.00615 |
